# Eco-Functional Epoxy Composites from Recycled ZnO and Tire Rubber: A Study on Breakdown Voltage Enhancement

**DOI:** 10.3390/ma19071373

**Published:** 2026-03-30

**Authors:** Bystrík Dolník, Vladimír Marcinov, Pavol Liptai, Miloš Matvija, Jakub Klimko, Dušan Oráč

**Affiliations:** 1 Department of Electric Power Engineering, Faculty of Electrical Engineering and Informatics, Technical University of Košice, Mäsiarska 74, 04200 Košice, Slovakia; bystrik.dolnik@tuke.sk; 2 Institute of Recycling and Environmental Technologies, Faculty of Materials, Metallurgy, and Recycling, Technical University of Košice, Letná 9, 04200 Košice, Slovakia; vladimir.marcinov@tuke.sk (V.M.); jakub.klimko@tuke.sk (J.K.); dusan.orac@tuke.sk (D.O.); 3 Institute of Materials, Faculty of Materials, Metallurgy, and Recycling, Technical University of Košice, Letná 9, 04200 Košice, Slovakia; milos.matvija@tuke.sk

**Keywords:** high-voltage insulation, epoxy resin, waste recycling, tire rubber, ZnO, breakdown voltage, circular economy

## Abstract

The increasing demand for sustainable materials in electrical engineering has encouraged the substitution of conventional fillers in epoxy insulation with recycled industrial by-products. This study investigates the potential use of waste tire rubber particles and zinc oxide recovered from electric arc furnace dust as eco-friendly fillers for epoxy resins in high-voltage insulation applications. Four material variants were fabricated: pure epoxy, epoxy with 10 wt% ZnO (0.7 mm thickness), epoxy with 10 wt% tire rubber (0.9 mm thickness), and epoxy with 20 wt% tire rubber (0.9 mm thickness). The breakdown voltage of each composite was measured under AC voltage. Results indicate that filler type and concentration influence breakdown behavior within each thickness group. The 10 wt% ZnO-filled epoxy exhibited a moderate enhancement in breakdown voltage compared with pure epoxy of the same thickness, consistent with interfacial modifications commonly observed in oxide-filled epoxy systems. Conversely, tire rubber fillers led to reduced breakdown performance, likely due to increased dielectric heterogeneity introduced by the elastomeric phase. No direct comparison between ZnO- and rubber-filled systems was performed due to differences in manufacturable sample thickness. The findings contribute to the evaluation of recycled fillers in dielectric composite systems within a circular-economy framework.

## 1. Introduction

The transition toward a circular economy has become a critical global priority, emphasizing the recovery and reuse of industrial waste materials to reduce environmental impact and resource depletion [1,2]. The automotive and metallurgical sectors, among the largest producers of waste, generate significant quantities of end-of-life tires and electric arc furnace (EAF) dust, both of which contain materials with substantial reuse potential [3,4,5,6]. In recent years, the concept of resource circularity has been increasingly integrated into materials engineering, driving innovation in recycling technologies and sustainable product design [7,8]. Moreover, comprehensive approaches involving life-cycle assessment (LCA) and selective recovery of functional components have demonstrated that such recycling routes can substantially reduce the carbon footprint of industrial systems [9,10,11].

Polymer-based materials, particularly epoxy resins, play a vital role in the electrical and electronics industries due to their excellent dielectric properties, mechanical strength, and thermal stability [12,13,14]. However, their thermosetting nature poses substantial challenges for recycling and reuse [15,16]. Recent advances have explored chemical depolymerization, hot-press reconfiguration, and ionic-liquid-assisted degradation as promising methods for recovering epoxy systems [17,18,19,20]. These approaches not only improve material recyclability but also contribute to reducing solid waste in high-tech sectors such as electrical insulation manufacturing [13,15,21]. In addition, sustainable polymer composites incorporating recycled fillers, such as nanofibers, natural polymers, and industrial residues, have shown potential for high-performance insulation and structural applications [11,12,22,23,24,25].

Parallel to polymer recycling, considerable research attention has been directed toward valorizing waste rubber, particularly from end-of-life vehicle tires, as a secondary resource [2,4,14,16,17]. Recycled tire rubber has been utilized as a modifier or filler in a range of applications, including concrete, asphalt, and polymer composites, improving mechanical flexibility, toughness, and thermal insulation [4,17,26]. Furthermore, environmental and health assessments of rubber-derived materials indicate that, when properly processed, they meet safety criteria for use in construction and civil engineering [18]. Incorporating tire rubber into polymer matrices not only supports waste reduction but also enhances energy absorption and impact resistance, properties valuable in both structural and electrical components [26,27,28]. Some studies have even demonstrated the feasibility of using rubber–soil mixtures for seismic isolation of electrical transformers, highlighting the synergy between recycled materials and electrotechnical infrastructure [28].

Among metal-based waste products, EAF dust represents a foremost source of recoverable zinc oxide (ZnO). This by-product, if untreated, poses severe environmental risks due to its heavy metal content, yet it can be efficiently processed into high-purity ZnO through hydrometallurgical or pyrometallurgical routes [29,30]. Recent works have demonstrated that ZnO obtained from recycled EAF dust can be effectively utilized in producing varistors and other ceramic components, exhibiting favorable electrical and structural characteristics [29,30,31]. The optimization of such recovery processes supports both sustainable metallurgy and the circular use of materials in electronic and power applications [31,32]. ZnO is also an established functional filler in polymer composites, improving dielectric strength, thermal conductivity, and partial discharge resistance–properties essential for high-voltage insulation materials [22,23,31].

While separate research streams have explored the recycling of epoxy resins, rubber particles, and ZnO-based fillers, limited studies have investigated their combined use to develop environmentally sustainable electrical insulation systems. Integrating these waste-derived components within an epoxy matrix could simultaneously address ecological concerns and advance functional performance in high-voltage engineering. The resulting composite insulation material may exhibit modified dielectric strength, breakdown behavior, and interfacial polarization characteristics, potentially offering a green alternative to conventional synthetic fillers [7,19,21,22,31].

According to our findings from the literature review, there are very few, if any, publications that address materials such as epoxy, waste ZnO fillers, and waste rubber for potential use in high-voltage technology. Based on these findings, the present study explores the integration of recycled zinc oxide and tire rubber powders as fillers in epoxy resin composites intended for high-voltage insulation applications. The investigation compares the dielectric breakdown strength of pure epoxy, epoxy with 10 wt.% ZnO, epoxy with 10 wt.% tire rubber, and epoxy with 20 wt.% tire rubber. The results provide insight into the influence of inorganic and organic recycled fillers on the electrical strength of epoxy-based insulating materials, supporting the transition toward sustainable and circular solutions in high-voltage insulation systems.

Therefore, the present study investigates the potential use of waste tire rubber and ZnO recovered from EAF dust as fillers in epoxy resin to develop a sustainable insulation material suitable for high-voltage applications. Four formulations were examined: pure epoxy, epoxy with 10 wt.% ZnO filler, epoxy with 10 wt.% tire rubber, and epoxy with 20 wt.% tire rubber. The breakdown voltage of each sample was measured to evaluate the influence of the waste-derived fillers on dielectric performance, providing insights into their suitability for environmentally conscious high-voltage insulation systems.

The remainder of this paper is organized as follows: Section 2 (Materials and Methods) describes the preparation and characterization of recycled ZnO and waste tire rubber fillers, the fabrication of epoxy composite samples, and the procedure for AC breakdown voltage testing using VDE electrodes. Section 3 (Results and Discussion) presents the measured breakdown voltages, statistical evaluation using the two-parameter Weibull distribution, and analysis of the influence of recycled fillers on dielectric strength. Section 4 (Conclusions) summarizes the key findings.

## 2. Materials and Methods

### 2.1. Filler Material Characterization and Preparation

#### 2.1.1. ZnO Filler Preparation

The ZnO material used in this study was obtained from the hydrometallurgical recycling of electric arc furnace dust. The recycling process comprised several steps: neutral leaching of EAF dust in distilled water, alkaline leaching using ammonium carbonate, multi-stage pressure filtration, cementation of impurities with zinc dust, and calcination of the final product. The recycling procedure was carried out in a semi-industrial facility at the Institute of Recycling Technologies.

The chemical composition of the recycled ZnO powder was determined by X-ray fluorescence (XRF) analysis using a Shimadzu 7000P spectrometer (Shimadzu, Kyoto, Japan). The results (Table 1) indicate that zinc is the predominant element, accounting for approximately 96.3 wt.%, confirming the high purity of the recovered ZnO. Minor amounts of sulfur, silicon, calcium, potassium, phosphorus, and trace metals such as iron, copper, vanadium, chromium, and manganese were also detected. These impurities originate from residual components of the EAF dust and the leaching reagents used during the recycling process.

After calcination, ZnO flakes were milled and sieved. The fraction smaller than 0.5 mm was dried at 105 °C for 24 h at atmospheric pressure and subsequently cooled in a desiccator before incorporation into the epoxy resin.

#### 2.1.2. Rubber Filler Preparation

**Figure 1:**
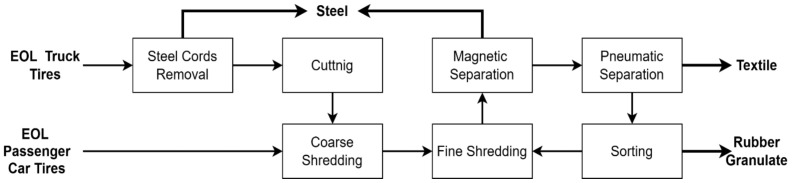
End-of-life (EOL) tire recycling flowsheet.

Recycled rubber was supplied by AVE SK, LLC company, operating in the Kechnec industrial park, Slovakia The material originated from mechanically processed end-of-life (EOL) tires recycled at ambient temperature. The recycling process consisted of tire cutting, followed by coarse and fine grinding, magnetic separation to remove steel components, and pneumatic separation to eliminate textile fibers, yielding rubber granulate as the final product (Figure 1).

We sieved the rubber granulate before further use, selecting the fraction below 1 mm. After sieving, the fraction obtained was dried at 60 °C for 48 h under atmospheric pressure. Once the weight reached a constant value, we stopped the drying process and continued with the next step of sample preparation.

### 2.2. Sample Preparation

A commercial-grade epoxy resin was used as the matrix material. The resin was mixed with the corresponding hardener at a mass ratio of 70:30 and stirred for 15 min at 250 rpm. The filler materials were then incorporated as follows: 10 wt.% ZnO and 10 or 20 wt.% rubber granules. The mixtures were homogenized by stirring for an additional 15 min at 250 rpm. To remove entrapped air, the mixtures were degassed in a vacuum chamber and subsequently poured into silicone molds. Curing was performed in a pressure chamber at 350 kPa for 14 days, followed by post-curing at atmospheric pressure for an additional 7 days. The cured samples were then machined to a constant thickness using a CNC milling machine and polished on both sides up to 5000 grit. The manufactured samples were then cut to size suitable for breakdown voltage testing using AC power frequency.

### 2.3. Methods

#### 2.3.1. Microscopy

Light microscopy (LM) was performed using an Olympus Vanox-T microscope (Olympus, Tokyo, Japan). We prepared samples for LM observation using standard metallographic procedures, including fine grinding (up to P4000 grit), two-step polishing (diamond paste followed by an oxide polishing suspension), and final cleaning in distilled water and methanol. To evaluate particle and/or microstructural feature size distributions in the analyzed samples, we used statistical image analysis (Olympus AnalySIS, start 5.1). Scanning electron microscopy (SEM) was performed using a Tescan MIRA3 microscope (Tescan, Brno, Czech Republic) equipped with a secondary electron detector. Both polished cross-sections and fracture surfaces were examined to assess the dispersion state of the fillers and the filler–matrix interfaces. We also used the same instrument for energy-dispersive X-ray spectroscopy (EDX) to analyze the elemental composition of the recycled fillers and to identify inorganic constituents and potential contaminants originating from the tire recycling process.

#### 2.3.2. AC Dielectric Breakdown Voltage Measurement

We used a progressive stress test protocol according to [33], or a short-time (rapid-rise) test [34]. An increasing voltage with a power frequency of 50 Hz is applied to each sample. We chose the method of applying the voltage by continuously increasing it with time, from zero voltage until dielectric failure of the test sample occurs. The rate-of-rise of the applied voltage is 1 kV/s. The result of the progressive stress test is the breakdown voltage on each sample.

In order to ensure that the tests were carried out under the same conditions, we selected identical samples with the same history before testing. We also ensured that the rate of voltage rise, sample thicknesses, electrode material, and other physical factors affecting the breakdown electric field when an alternating voltage is applied were the same during testing. The experiment was conducted at a temperature of 23 °C to 24 °C and a relative humidity of 47 ± 2%.

**Figure 2:**
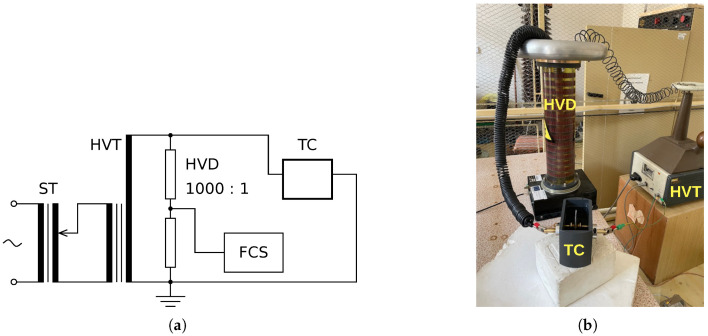
Experimental test measuring circuit (**a**) and view of the high-voltage part of the measuring equipment (**b**): ST—setting transformer, HVT—high-voltage transformer, HVD—high-voltage divider, TC—test cell, FCS—four channels scope.

The experiment was conducted using a high-voltage test circuit. The electrical diagram of the measuring circuit, depicted in Figure 2, consists of a setting transformer (ST) with overcurrent protection, a high-voltage transformer (HVT), a high-voltage divider (HVD) up to 100 kV with a division ratio of 1000:1, a test cell (TC), epoxy samples and epoxy samples with ZnO filler or waste tire rubber particles, silicone insulating oil, and four channels scope (FCS) 1 GSa/s, 200 MHz connected.

The test sample was placed in a test cell between two VDE electrodes [35] placed opposite each other. We used VDE electrodes because a test cell equipped with them was available. The dominant material of the tested samples is epoxy, a relatively hard material. When inserting the sample between the test electrodes, we took care to avoid excessive mechanical stress because of the pressure between the electrodes. To prevent surface discharges and possible flashover on the sample surface, we used silicone insulating oil commonly used for high-voltage equipment. Thus, the electrodes and the test sample were immersed in the oil. The impact of changes in oil quality on test results was minimized through frequent oil changes. Each test on the samples was recorded using FCS until sample breakdown to determine the exact breakdown voltage. The FCS sampling rate was set to 5 kHz. The total time record of the applied voltage was saved in CSV format for further processing. The actual thickness of the sample was measured after the test in the immediate vicinity of the puncture site using a micrometer with a 0.001 mm smallest reading at room temperature. Electrical breakdowns evaluated using the Weibull distribution were considered only on samples whose thickness varied by no more than ±0.02 mm. In this way, five samples from each thickness group were selected for evaluation using the Weibull distribution.

**Figure 3:**
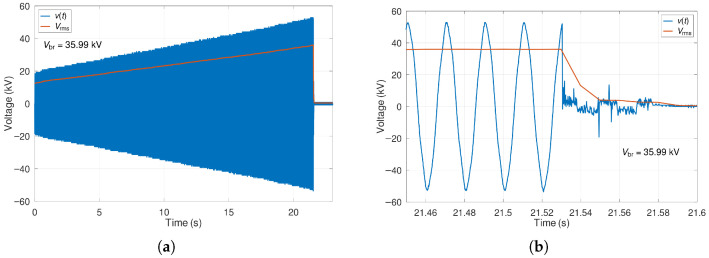
Graphs generated by the script: (**a**) waveform of the applied voltage; (**b**) enlargement at the moment of sample breakdown. The orange line represents the trend of the numerically calculated RMS values in each half-cycle of the applied voltage.

To determine the breakdown voltage of the samples, we used a script in the scientific programming language GNU Octave version 8.0.0 on the Ubuntu 20.04 LTS operating system. The script read all CSV records sequentially and, using a numerical method, calculated the root-mean-square (RMS) voltage in each half-cycle of the applied voltage. At the same time, the script generated two graphs for each sample. The first graph shows a time record of the instantaneous applied voltage and the calculated RMS voltage for each half-cycle. At the same time, the script writes the breakdown voltage value to the first image. The second graph is an enlargement of the moment of the sample breakdown and helps check the total breakdown. Figure 3 shows an example of graphs generated by the script.

#### 2.3.3. Data Processing and Weibull Statistical Analysis

To evaluate the electrical breakdowns of the samples, we used the Weibull distribution, which was implemented using a custom-written script in Python 3. The Weibull distribution is the most common for solid insulation because of its wide applicability. The above distribution can be described by two parameters, similar to the normal distribution described by the mean and standard deviation [36].

The adequacy of the selected Weibull distribution representing the distribution data set can be verified by plotting the data points on a special probability plot. If there is a good fit to the distribution, the result will be a straight line plot. The cumulative density function for the two-parameter Weibull distribution describes Equation (1)

(1) $$F\left(V;\alpha ,\beta \right)=1-\exp\left[-{\left(\frac{V}{\alpha }\right)}^{\beta }\right],$$

where *V* is the measured variable, in our case it is breakdown voltage, F(V) is the probability of failure (breakdown) at a voltage less than or equal to *V*, α is the scale parameter and is positive, and β is the shape parameter and is positive. The failure probability F(V) is zero at V=0 (*V* represents the applied voltage). In our case, the failure probability of the electrical breakdown of the sample increases continuously with increasing *V*. Then, with increasing voltage, the failure probability approaches certainty, i.e., F(∞)=1. The scale parameter α represents the voltage for which the probability of failure is 0.632 based on 1−1/e, where e is Euler’s number. It is analogous to the mean of a normal distribution. The units of α are the same as *V*. The shape parameter β is a measure of the range of breakdown voltages. The larger the β, the smaller the range of breakdown voltages. It is analogous to the inverse of the standard deviation of the normal distribution [33].

The correlation coefficient for testing the adequacy of the Weibull distribution is calculated using the formula given in the IEC 62539 [33], namely:

(2) $$F\left(i,n\right)=\frac{i-0.44}{n+0.25}\times 100\%,$$

with the following transformations:

(3) $$Y\left(i\right)=\ln\left(V_{\mathrm{br}}\right),$$

(4) $$X\left(i\right)=\ln\left\{-\ln\left[1-\frac{F(i,n)}{100}\right]\right\}.$$

The correlation coefficient r is then determined using the least squares regression method, i.e., the Pearson correlation coefficient between $X_{i}$ and $Y_{i}$. To determine whether the data points fit well with a two-parameter Weibull distribution, we used the procedure outlined in the [33] standard. The original data presented in the study, as well as the scripts used for data processing, are publicly available in [37].

**Figure 4:**
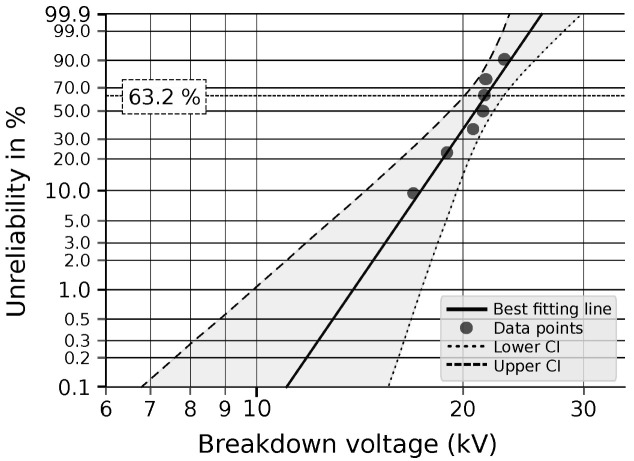
Weibull plot with confidence intervals based on fictitious data.

In Figure 4, an illustrative Weibull probability plot with confidence bounds is presented to demonstrate the fitting procedure. The solid line represents the best-fit Weibull distribution obtained by the median rank regression method, while the dashed lines denote the lower and upper confidence bounds (2σ) calculated using the Monte Carlo Pivotal Bounds method according to IEC 62539. The individual data points correspond to ficticious breakdown voltages. Numerical values of the confidence limits are not explicitly reported, as the bounds are evaluated graphically in accordance with IEC 62539.

The Weibull scale (α) and shape (β) parameters were estimated using the Median Rank Regression (MRR) as primary point estimates. To quantify the uncertainty and robustness of these estimates, a non-parametric bootstrap procedure was performed, in which multiple resampled datasets were generated, and the Weibull parameters were re-estimated for each case. The average values obtained from the bootstrap resampling, together with percentile-based 95% confidence intervals, were used to characterize the statistical variability of the parameter estimates.

The reliability of the shape parameter estimated from the original dataset was assessed by verifying whether it falls within the bootstrap-derived confidence interval, indicating the absence of systematic estimation bias. Occasional failed bootstrap fits were observed for resampled datasets with low variability and were found to have a negligible effect on the overall results. Due to the computational cost of the bootstrap procedure, calculations were parallelized across all available CPU cores, significantly reducing computation time without affecting numerical accuracy.

#### 2.3.4. Dielectric Spectroscopy Measurements

We characterized the dielectric properties of the prepared samples by measuring the relative permittivity ($\varepsilon_{r}$) and dielectric loss factor (tanδ) using an Agilent E4980A precision LCR meter (Agilent, Santa Clara, CA, USA). The measurements were performed over a frequency range from 20 Hz to 2 MHz at a controlled temperature of 23 ± 1 °C and a relative humidity of 30%. The measurements were carried out using a parallel-plate electrode configuration with stainless steel electrodes, with the sample placed between two flat electrodes to form a capacitor. Before the measurements, we calibrated the instrument according to the manufacturer’s recommended procedure, including open-circuit and short-circuit calibrations of the measurement chain. We calculated the relative permittivity as the ratio of the sample capacitance to the corresponding geometrical capacitance, determined from the sample thickness and electrode area. The dielectric loss factor was directly obtained from the instrument output. For each sample, we performed five independent measurements, and the values presented in the frequency-dependent plots represent the average obtained from these measurements.

## 3. Results and Discussion

### 3.1. Microstructural Characterization of Recycled Rubber-Epoxy Composites

**Figure 5:**
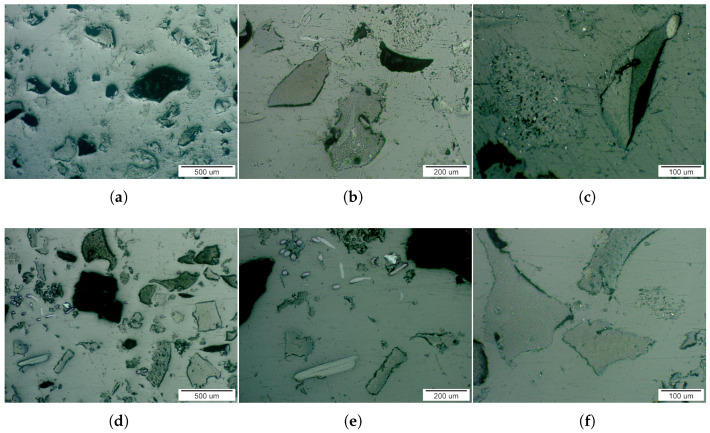
Microstructures of composite materials consisting of an epoxy matrix with 10 wt.% tire rubber filler (**a**–**c**) and 20 wt.% tire rubber filler (**d**–**f**), documented by LM at different magnifications.

Microstructural analysis revealed the morphology and spatial distribution of recycled tire rubber particles within the epoxy matrix for composites containing 10 wt.% and 20 wt.% filler, as shown in Figure 5.

**Figure 6:**
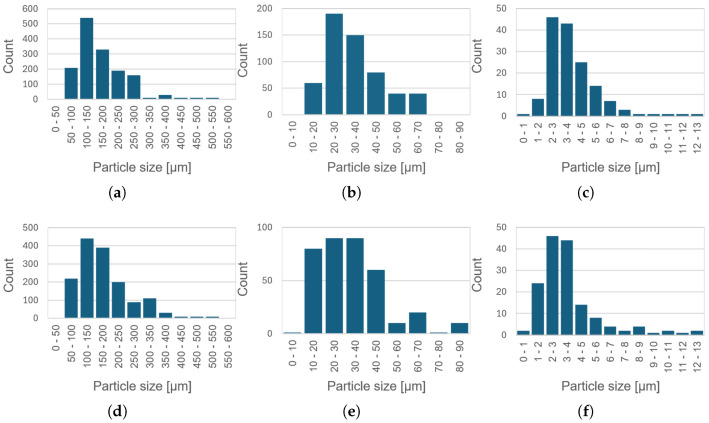
Particle size distributions of composite containing 10 wt.% recycled filler: coarse particles (**a**), smaller particles (**b**), and fine particles (**c**); and composite containing 20 wt.% recycled filler: coarse particles (**d**), smaller particles (**e**), and fine particles (**f**).

Light microscopy observations indicated the presence of particles with a broad range of sizes and morphologies. Coarse particles were predominantly sharp-edged and irregular or polyhedral in shape, whereas smaller particles exhibited oval or rod-like morphologies. Fine particles were mainly globular and exhibited a metallic-like appearance, suggesting the presence of inorganic constituents. Quantitative image analysis revealed a multimodal particle-size distribution at both filler concentrations (Figure 6). The average particle sizes of the coarse, smaller, and fine fractions are summarized in Table 2. The similarity of particle size distributions for the 10 wt.% and 20 wt.% composites indicate that increasing filler content primarily affects particle number density rather than particle size. Filler dimensions represent a critical parameter governing composite performance, as they influence interfacial area, dispersion homogeneity, local electric field distribution, and the probability of forming conductive networks. Previous studies have demonstrated that filler lateral size and aspect ratio can significantly modify transport properties and interfacial effects in polymer composites [38]. Although that work addressed thermal transport in graphene systems, it highlights the broader structure-property relationship associated with filler dimensions, which is also relevant for dielectric composites.

**Figure 7:**
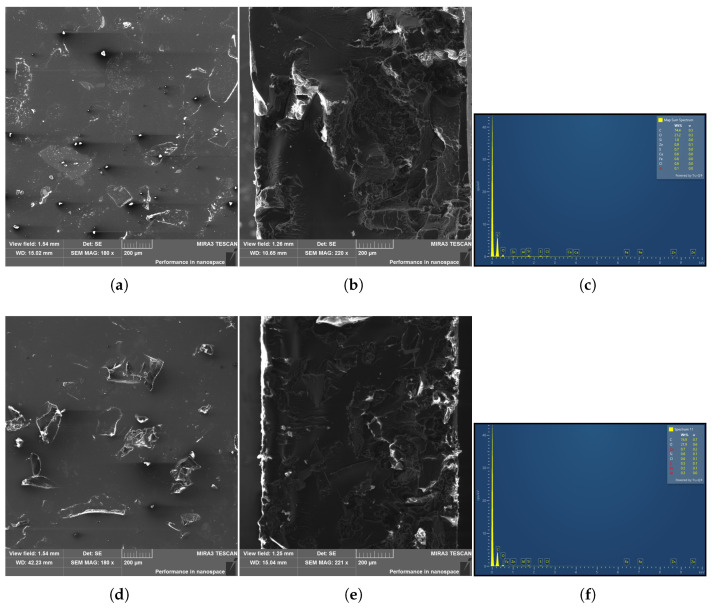
Microstructures of composite materials consisting of an epoxy matrix with 10 wt.% tire rubber filler: polished state (**a**), fracture surface (**b**) documented by SEM, and EDX spectrum of the analyzed area (**c**); and composite materials consisting of an epoxy matrix with 20 wt.% tire rubber filler: polished state (**d**), fracture surface (**e**) documented by SEM, and EDX spectrum of the analyzed area (**f**).

SEM observations of polished and fracture surfaces (Figure 7) confirmed a generally homogeneous dispersion of recycled filler particles within the epoxy matrix, although local clustering of fine particles was occasionally observed. The fracture surface morphology suggests predominantly brittle matrix behavior with localized filler–matrix debonding, reflecting the heterogeneous nature of the recycled rubber particles. EDX analysis revealed a complex elemental composition of the recycled fillers, dominated by carbon and oxygen, with minor amounts of Zn, S, Si, Ca, Fe, Cl, and Al, (Table 3) consistent with typical tire rubber formulations and residual inorganic additives. The elemental composition was very similar for both filler concentrations, indicating comparable chemical characteristics of the recycled material.

From a dielectric perspective, the observed heterogeneous particle size distribution, irregular particle morphology, and chemically complex filler composition are expected to increase interfacial area and dielectric heterogeneity within the composite. Such features are commonly associated with enhanced interfacial polarization and increased dielectric losses in polymer composites. While the present microstructural analysis does not allow a direct quantification of specific interfacial mechanisms, it establishes an essential structural framework for the subsequent interpretation of the dielectric permittivity, dielectric loss, and AC breakdown measurements presented in the following sections.

Detailed SEM-EDX characterization of the recycled ZnO material has been previously reported in our earlier work (Sections 2.1 and 2.2 in [31]), and is therefore not repeated here.

### 3.2. AC Breakdown Voltage Performance and Weibull Statistical Analysis

The breakdown performance of the epoxy composites was evaluated under AC voltage stress, and the statistical analysis of failure data was performed using Weibull distribution fitting implemented in Python. The parameters α (characteristic breakdown voltage) and β (shape factor) were determined for all tested configurations, providing a quantitative measure of dielectric endurance and data dispersion.

**Figure 8:**
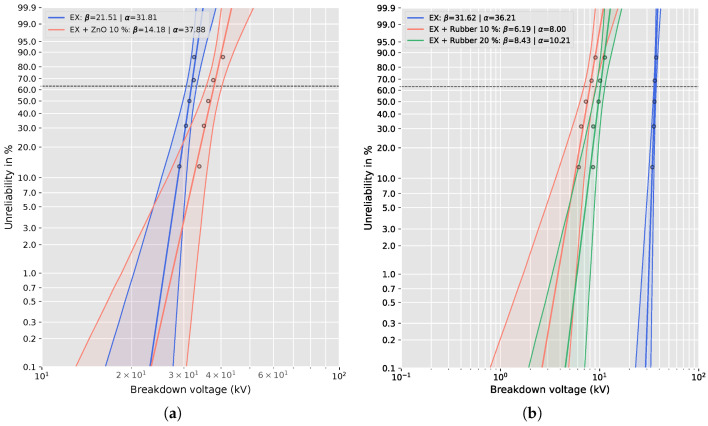
Weibull probability plots for the breakdown voltage (α) of the epoxy composites with and without recycled fillers: (**a**) pure epoxy and epoxy + 10 wt% ZnO; (**b**) pure epoxy, epoxy + 10 wt%, and epoxy + 20 wt% tire rubber.

Figure 8 presents the Weibull probability plots for the breakdown voltage of the epoxy composites with and without recycled fillers. The slope of each line corresponds to the shape parameter (β), while the intercept provides the characteristic breakdown voltage (α) at 63.2% cumulative failure probability.

The pure epoxy samples exhibit a steep slope (β = 21.51 and α = 31.62), indicating high uniformity of breakdown events and stable dielectric properties. Incorporation of 10 wt% ZnO filler (in Figure 8 marked as Ex + ZnO 10%) slightly reduced the slope (β = 14.18), implying increased data scatter, but the higher α value (37.88 kV) confirms improved dielectric endurance.

In contrast, rubber-filled composites show significantly flatter slopes (β = 6.19 for 10 wt% and 8.43 for 20 wt%), suggesting non-uniform electric field distribution and premature failure initiation at filler-matrix interfaces. These findings are consistent with the observed 4.53 times and 3.55 times reductions in breakdown voltage compared to pure epoxy, confirming the detrimental influence of elastomeric inclusions on the electrical integrity of the insulation system. In Figure 8, the dashed line represents the percentage value of the scale parameter for which the probability of failure is 63.2%.

Table 4 summarizes the obtained Weibull parameters, along with the relative change in characteristic breakdown voltage (α) compared to the reference epoxy sample. For samples containing ZnO filler (0.7 mm thickness), the incorporation of 10 wt% ZnO led to an increase in α from 31.81 kV to 37.88 kV, representing a 19% enhancement in breakdown voltage.

This improvement is consistent with the dielectric behavior commonly reported for oxide-filled epoxy systems, in which modifications to the local electric field distribution, interfacial structure, and charge dynamics at the filler-matrix interfaces may influence breakdown processes [39]. In the present case, the interpretation is supported by the experimentally observed dielectric response, including changes in permittivity and increased dielectric losses (see the corresponding graphs in the following subsection), which suggest enhanced interfacial polarization effects rather than providing direct evidence of a specific microscopic mechanism.

In contrast, the addition of waste tire rubber particles (0.9 mm samples) significantly reduced the breakdown performance. The α parameter decreased from 36.21 kV for the reference epoxy to 8.00 kV and 10.21 kV for 10 wt% and 20 wt% rubber-filled samples, respectively, corresponding to a 4.53-fold and 3.55-fold reduction.

This pronounced decline in breakdown performance is consistent with the microstructural characteristics identified in the rubber-filled composites. Light microscopy and SEM observations reveal a multimodal particle-size distribution, irregular particle morphology, and heterogeneous filler-matrix interfaces, along with occasional local clustering of fine particles. Such structural heterogeneity increases dielectric inhomogeneity within the composite and may result in a non-uniform electric field distribution under AC stress. Furthermore, the relatively large particle fraction (<1 mm) used in the present study is comparable to the sample thickness (0.9 mm), suggesting that the rubber phase occupies a substantial portion of the electrical path. Since the intrinsic dielectric strength of elastomeric materials is generally lower than that of the epoxy matrix, this geometrical and material contrast can reduce the effective breakdown resistance of the composite.

The β parameter also decreased notably for the 10 wt% rubber composite (β=6.19), indicating higher statistical scatter and reduced homogeneity of the breakdown process. The dielectric strength given in Table 4 is calculated as the quotient of the breakdown voltage (α) and the distance between the electrodes (sample thickness), over which the voltage is applied.

Although only a single rubber particle size fraction (<1 mm) was investigated in the present study, microstructural analysis did not reveal the formation of continuous conductive pathways across the sample thickness. SEM observations indicate heterogeneous dispersion with occasional local clustering rather than interconnected filler networks. Furthermore, the frequency-dependent dielectric spectra do not exhibit features characteristic of percolation-type conduction. Therefore, we interpret the observed deterioration in breakdown strength as a consequence of increased structural and electrical heterogeneity rather than the establishment of macroscopic conductive networks. A systematic evaluation of different particle size fractions remains a subject for future investigation.

All correlation coefficients in Table 4 exceed the critical correlation coefficient, which for five samples is approximately $r_{\mathrm{crit}}=0.896$. This result indicates a good agreement with the two-parameter Weibull distribution [33]. Hence, the data points can be regarded as well described by this distribution. We emphasize here that the dielectric strength values presented in Table 4 are not intended for direct comparison between samples of different thicknesses. Although the breakdown voltage was normalized to sample thickness and expressed as dielectric strength, it is well known that the breakdown voltage-thickness relationship may be non-linear and that differences in breakdown mechanisms can persist even after such normalization.

Therefore, all comparisons and interpretations in this study are strictly limited to samples of identical thickness. We evaluated the effect of recycled ZnO filler by comparing epoxy-based samples with a thickness of 0.7 mm against the corresponding pure epoxy reference of the same thickness. We assessed the influence of tire rubber filler exclusively within the 0.9 mm thickness group. We always calculated the relative change in the Weibull scale parameter α with respect to the pure epoxy sample of the same thickness. For this reason, we draw the reader’s attention to the fact that the authors did not compare samples with different thicknesses in the conclusion, and that the inclusion of multiple thicknesses in Table 4 serves only to provide a compact overview of the experimental data set.

**Figure 9:**
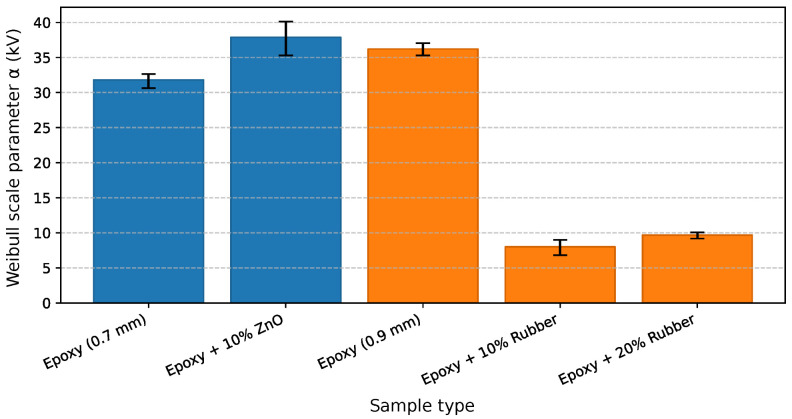
Weibull scale parameter comparison—characteristic breakdown voltage α for epoxy-based composites with different fillers and thicknesses. Error bars represent 95% confidence intervals obtained by a non-parametric bootstrap resampling method applied to the breakdown voltage datasets.

**Figure 10:**
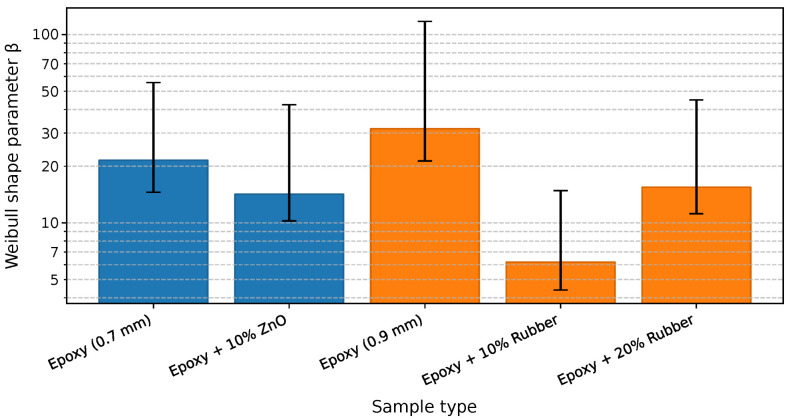
Weibull shape parameter β for epoxy-based composites with different fillers and thicknesses. Error bars indicate 95% bootstrap confidence intervals. Note the logarithmic scale of the vertical axis.

To provide a statistically meaningful comparison of the dielectric performance, the characteristic breakdown voltage α and shape parameter β are presented separately in Figure 9 and Figure 10. The error bars represent 95% confidence intervals obtained by a non-parametric bootstrap resampling method, reflecting the uncertainty associated with parameter estimation for the limited sample size (n=5). Importantly, all comparisons are performed exclusively between samples of identical thickness to avoid thickness-related bias.

For the 0.7 mm thick samples, a direct comparison between the neat epoxy and the epoxy composite containing 10 wt% ZnO shows a clear enhancement in dielectric strength. The Weibull scale parameter α increases from 31.81 kV for the neat epoxy to 37.88 kV for the ZnO-filled system, corresponding to an improvement of approximately 19%. The relatively narrow confidence intervals of α indicate a robust and reproducible improvement in breakdown performance. At the same time, the Weibull shape parameter β remains at a comparable level (21.51 for neat epoxy and 14.18 for the ZnO composite), indicating that the increase in breakdown strength is not accompanied by a substantial increase in data dispersion.

For the 0.9 mm thick samples, the effect of waste tire rubber fillers is evaluated by comparing the neat epoxy with composites containing 10 wt% and 20 wt% rubber. In this thickness group, one can observe a pronounced deterioration in dielectric strength. The Weibull scale parameter α decreases significantly for both rubber-filled systems, indicating a substantial reduction in characteristic breakdown voltage. We believe that this degradation is primarily associated with increased dielectric inhomogeneity and the formation of weak interfacial regions introduced by the elastomeric inclusions, which can locally intensify the electric field and facilitate premature breakdown.

The corresponding Weibull shape parameter β for the 0.9 mm samples shows an apparent increase in breakdown variability in the rubber-filled composites, particularly in the 10 wt% rubber system (β=6.19), compared to the neat epoxy reference. We assume this behavior reflects increased sensitivity to localized defects and microstructural heterogeneity, likely related to poor interfacial adhesion between the epoxy matrix and the rubber particles. Despite the relatively broad confidence intervals of β, the median rank regression estimates remain within the bootstrap confidence bounds for all samples, confirming the statistical consistency of the analysis.

### 3.3. Dielectric Response of Epoxy Composites

**Figure 11:**
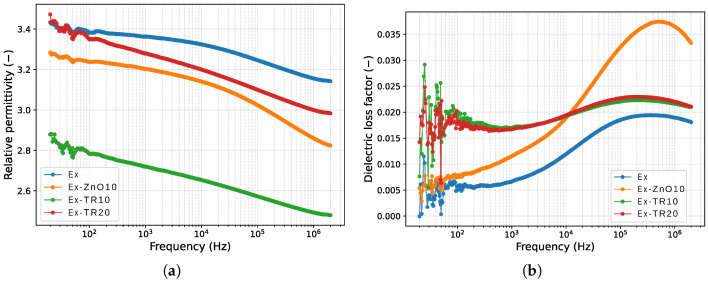
Frequency dependence of (**a**) dielectric constant ($\varepsilon_{r}$) and (**b**) dielectric loss factor (tan δ) for neat epoxy and epoxy composites filled with 10 wt% ZnO, 10 wt% tire rubber (TR), and 20 wt% tire rubber, measured at room temperature in the frequency range from 20 Hz to 2 MHz.

To complement the AC breakdown strength results and to provide a broader dielectric characterization, we measured the dielectric constant ($\varepsilon_{r}$) and dielectric loss factor (tanδ) of all investigated materials over a frequency range from 20 Hz to 2 MHz at room temperature. The frequency dependences of $\varepsilon_{r}$ and tanδ are shown in Figure 11.

All samples exhibit a similar qualitative behavior, characterized by a gradual decrease in permittivity with increasing frequency, which is typical of epoxy-based dielectric systems and is commonly attributed to the limited ability of dipolar and interfacial polarization mechanisms to respond to a rapidly alternating electric field. At 50 Hz, the relative permittivity of the neat epoxy reference is approximately 3.4, which is consistent with typical values reported in the literature for cured epoxy resins. The epoxy composite filled with 10 wt% ZnO shows a slightly lower permittivity of about 3.25, while the composite containing 10 wt% tire rubber exhibits the most pronounced decrease, reaching approximately 2.8. In contrast, the epoxy composite with 20 wt% tire rubber shows a permittivity of about 3.39, comparable to that of the neat epoxy. A similar frequency-dependent decrease in relative permittivity and a non-monotonic behavior of dielectric losses have been reported for epoxy composites filled with ZnO and TiO_2_ nanoparticles, where interfacial polarization at the epoxy-filler boundaries plays a significant role in determining the dielectric response [40,41,42,43].

The dielectric loss factor increases with frequency for all materials and reaches a maximum in the range from 220 kHz to 500 kHz, after which no further increase is observed within the investigated frequency window. The maximum values of tanδ lie in the range from 0.019 to 0.037, with the lowest dielectric losses observed for the neat epoxy (≈0.019). Slightly higher values are measured for the epoxy composites filled with 10 wt% and 20 wt% tire rubber (≈0.022), while the highest dielectric loss factor is observed for the epoxy + 10 wt% ZnO composite (≈0.037).

XRF analysis of the recycled ZnO revealed a Zn content of 96.3 wt%, with the remaining approximately 3.7 wt% consisting of minor elements, predominantly S (1.344 wt%), Si (0.793 wt%), Ca (0.519 wt%), K (0.399 wt%), P (0.245 wt%), and Fe (0.243 wt%). Such secondary constituents may influence dielectric behavior through modified interfacial polarization or local conductivity effects. However, the frequency-dependent dielectric measurements in the present study do not show pronounced conductivity-driven losses or anomalous dispersion. The dielectric response of the ZnO-filled composite remains consistent with interfacial effects typically reported for oxide-filled epoxy systems, suggesting that the overall filler-matrix interaction dominates over the contribution of minor impurity phases within the investigated composition range.

The occurrence of a maximum in the dielectric loss factor in the mid-frequency range (approximately from 220 kHz to 500 kHz) can be qualitatively related to the microstructural characteristics of the recycled-filler composites. The multimodal particle size distribution, irregular particle morphology, and chemically heterogeneous nature of the tire rubber filler increase the overall interfacial area and dielectric heterogeneity within the epoxy matrix. Such structural features are known to promote interfacial (Maxwell-Wagner-Sillars) polarization processes, which typically manifest as enhanced dielectric losses and a loss peak at intermediate frequencies. Although no direct spectroscopic identification of individual relaxation processes is provided in the present study, the observed tanδ maximum is consistent with interfacial polarization effects associated with heterogeneous filler-matrix systems.

EDX analysis of the recycled tire rubber confirms its chemically heterogeneous nature, dominated by carbon in the range of approximately (74–75) wt% and oxygen in the range of approximately (21–22) wt%, with minor inorganic constituents including Zn, Si, S, Fe, Cl, and Al present at sub-wt.% to low-wt.% levels. These elements originate from typical tire formulations (e.g., ZnO activators, silica fillers, sulfur crosslinking agents, and residual steel components). The chemically heterogeneous nature of recycled rubber increases dielectric heterogeneity within the composite and may promote interfacial polarization; however, the measured dielectric spectra do not indicate excessive conductivity.

Surface modification strategies have been widely reported as effective approaches to improve filler-matrix compatibility and reduce agglomeration in polymer composites. For example, functionalization of nanocarbon fillers has been shown to enhance dispersion, interfacial bonding, and mechanical performance in epoxy systems [44]. Similarly, surface treatment of ceramic nanoparticles (e.g., amino-functionalization) significantly improved interfacial adhesion, curing behavior, and mechanical strength of epoxy nanocomposites [45]. Such modification strategies could be applied to recycled rubber particles to mitigate dielectric heterogeneity and improve breakdown performance.

The experimentally obtained dielectric parameters are in good agreement with representative literature data summarized in Table 5, both in terms of absolute magnitude and observed trends. In particular, the increased dielectric losses observed in the ZnO-filled epoxy are consistent with enhanced interfacial polarization, a phenomenon commonly reported for oxide-filled epoxy systems. In contrast, the relatively low losses of the neat epoxy and rubber-filled composites reflect their more insulating character within the investigated frequency range. These results provide additional context for interpreting the AC breakdown behavior discussed in the previous sections.

## 4. Conclusions

This study investigated the potential of using recycled ZnO powder recovered from electric arc furnace dust and waste tire rubber particles as fillers in epoxy-based insulation materials intended for high-voltage applications. We analyzed the breakdown voltage behavior of epoxy composites containing 10 wt% ZnO and rubber contents of 10 wt% and 20 wt%, and compared these results with pure epoxy. Breakdown testing, performed with VDE electrodes under controlled laboratory conditions, used the two-parameter Weibull distribution for statistical analysis, as recommended by IEC 62539.

The epoxy composite filled with 10 wt% recycled ZnO exhibited a 19% increase in breakdown voltage compared with pure epoxy. The Weibull scale parameter α increased from 31.81 kV for pure epoxy to 37.88 kV for the ZnO-filled composite, indicating enhanced dielectric strength. Although the shape parameter β decreased slightly from 21.51 to 14.18, suggesting greater variability in breakdown events, the overall improvement in electric strength confirms the beneficial effect of the recycled ZnO filler. We assume that the observed enhancement arises from the high purity of the recovered ZnO (96.3 wt%), which promotes interfacial polarization and charge trapping within the epoxy matrix.

In contrast, the incorporation of waste tire rubber particles led to a gradual reduction in breakdown voltage. The α parameters for composites with 10 wt% and 20 wt% rubber were 8 kV and 10.21 kV, respectively—corresponding to 4.53-fold and 3.55-fold decreases compared with pure epoxy. The lower shape parameters (β=6.19 and 8.43) indicate larger statistical dispersion and unstable dielectric behavior. The reduction in dielectric strength likely results from interfacial defects, carbon residues, and non-polar rubber domains that disrupt the uniformity of the electric field and facilitate early breakdown initiation.

Future research will focus on several key directions. We will explore hybrid filler systems combining recycled ZnO with small proportions of rubber to balance mechanical flexibility and electrical performance. In addition, future studies could benefit from advanced dielectric characterization techniques, such as impedance spectroscopy or thermally stimulated current measurements, which offer deeper insight into charge transport and interfacial phenomena relevant to breakdown behavior. Finite-element simulations of local electric field distributions within heterogeneous composites could provide deeper insights into breakdown initiation processes and the role of interfacial effects. Finally, we will perform long-term aging studies to assess the durability and reliability of the recycled-filler epoxy composites under realistic high-voltage operating conditions.

The present results provide an initial assessment of room-temperature electrical performance. The influence of thermal transitions, including the glass transition temperature, as well as a comprehensive evaluation of thermal stability, mechanical integrity, partial discharge resistance, and long-term aging behavior, remains a subject for future investigation before practical implementation in high-voltage insulation systems.

For the rubber-filled systems, the present results represent a baseline evaluation of untreated recycled material. Future optimization strategies may include surface modification of rubber particles to improve interfacial adhesion, the use of compatibilizers or coupling agents to reduce dielectric mismatch, the selection of narrower particle-size fractions, and hybrid filler systems combining rubber with oxide fillers such as ZnO. Implementing these strategies can reduce structural heterogeneity and improve the dielectric performance of the composites.

## Figures and Tables

**Table 1:** Chemical composition of ZnO powder determined by XRF analysis.

Elem. ^1^	Zn	S	Si	Ca	K	P	Fe	Cu	V	Cr	Mn
wt.%	96.295	1.344	0.793	0.519	0.399	0.245	0.243	0.057	0.05	0.03	0.019

^1^ Elem. is an acronym for element.

**Table 2:** Average particle size in the analyzed composite materials (µm).

Measured Particles	10% Fillers in Epoxy Matrix	20% Fillers in Epoxy Matrix
Coarse	170.1 ± 76.2	178.0 ± 83.2
Smaller	44.8 ± 13.5	43.3 ± 15.0
Fine	3.8 ± 1.6	3.4 ± 2.1

**Table 3:** Elemental composition of epoxy composites with recycled tire rubber fillers determined by EDX analysis (wt.%). Minor elements below the detection limit are not reported.

Element	10% Recycled Filler	20% Recycled Filler
C	74.4	74.9
O	21.2	21.9
Si	1.0	0.6
Zn	0.9	0.7
S	0.7	0.5
Ca	0.6	–
Fe	0.6	0.5
Cl	0.6	0.6
Al	0.1	0.3

**Table 4:** Weibull parameters of epoxy composites with recycled fillers (measured under AC voltage).

Sample ID	Filler Type and Concentration	Thickness (mm)	α (kV)	β (–)	Relative Change in α vs. Pure Epoxy	Correlation Coefficient (Pearson)	Dielectric Strength (kV/mm)
E-0.7	Pure epoxy (reference, 0.7 mm)	0.7	31.81	21.51	—	0.98587	45.44
E-ZnO10	Epoxy + 10 wt% ZnO (EAF dust)	0.7	37.88	14.18	+19%	0.95270	54.11
E-0.9	Pure epoxy (reference, 0.9 mm)	0.9	36.21	31.62	—	0.98062	40.23
E-R10	Epoxy + 10 wt% tire rubber	0.9	8.00	6.19	−77.4% (4.53× lower)	0.96073	8.89
E-R20	Epoxy + 20 wt% tire rubber	0.9	10.21	8.43	−71.8% (3.55× lower)	0.92787	11.34

**Table 5:** Representative literature values of dielectric constant ($\varepsilon_{r}$) and dielectric loss factor (tanδ) for neat epoxy and epoxy composites with various fillers at room temperature. Reported values depend on frequency, filler content and interface chemistry; therefore, the values listed here are indicative and given together with the corresponding experimental conditions ($\varepsilon_{r}$ and tanδ are representative at 1 kHz unless noted, RT—room temperature, LF—low frequency).

Material System	Filler Content	$\varepsilon_{r}$	tanδ	Conditions and Reference
Neat epoxy (DGEBA-based, cured)	–	3.2–3.8	≤0.03	Manufacturer data and broadband dielectric spectroscopy; RT [46]
Epoxy + ZnO (semiconducting/varistor particles)	≤50 vol%	3.5–6	$10^{-2}$–$10^{-1}$	Dielectric spectroscopy; moderate loading regime [47]
Epoxy + ZnO (high loading, near percolation)	>70 vol%	>10 (up to tens)	>0.1 (can exceed 1)	Strong interfacial polarization and conduction effects; low–mid frequency [47]
Epoxy + TiO_2_ (nanoparticles/nanowires)	1–10 wt%	3.8–5.0	0.02–0.08	Broadband dielectric spectroscopy; particle shape dependent; RT [46]
Epoxy + SiO_2_ (nano-silica, surface functionalized)	1–5 wt%	3.1–3.6	≤0.03	Insulating filler; small changes depending on dispersion and coupling agent [48]
Epoxy + rubber	10–25 vol%	3.7–4.3 (LF)	0.007–0.35	interfacial polarization and interfacial losses [49,50]

## Data Availability

Our original data presented in this study, as well as the scripts used to process the data, are publicly available in Mendeley at https://data.mendeley.com/datasets/4cb6rdm4w9/1 (accessed on 24 March 2026).

## Abbreviations

The following abbreviations are used in this manuscript:

AC	alternating current
CSV	comma-separated values
CNC	computer numerical control
EAF	electric arc furnace
EDX	energy-dispersive X-ray spectroscopy
Elem.	element
FCS	four channels scope
GNU	GNU’s Not Unix!
HVD	high-voltage divider
HVT	high-voltage transformer
IEC	International Electrotechnical Commission
LCA	life-cycle assessment
LF	low frequency
LM	light microscopy
LTS	long-term support
RMS	root-mean-square
RT	room temperature
SEM	scanning electron microscopy
ST	setting transformer
TC	test cell
ZnO	zinc oxide
XRF	X-ray fluorescence
